# Dr. Peebles Case of Hysterical Affection of the Eyes, with Obstinate Closure of the Lids

**Published:** 1842-08-27

**Authors:** 


*Case of Hysterical Affection of the Eyes, with obstinate Closure of the Lids.* By Dr. Peebles.—About ten years ago a lady, now twenty-seven, active and healthy, was attacked, the morning following a party, with intolerance of light, pain and watering of the eyes, and then complete closure of the lids, but without spasmodic action or distortion of any kind. She was unable to open her eyelids herself, and *no force which could be employed sufficed to do so,* until she was bled to ℥viij., when they opened spontaneously; but in forty-eight hours they closed anew, to be again opened by the same means. During two years and a half the attacks recurred at irregular intervals, especially in the right eye, which never remained for more than a week unaffected: and during this period, besides venesection and arteriotomy, acupuncture of the lids, electricity, and the moxa to the vertex, were employed with success. When the eyelids were soon opened, the conjunctiva appeared natural; but when the opening was delayed, this membrane appeared flocculent and granular, and discharged a wheyish, purulent fluid. Almost every medicine in use, alteratives, tonics, antispasmodics, narcotics, have been tried, along with sea-bathing and voyages and travelling; and almost all the eminent medical men in Dublin, and several in London, have been consulted without the slightest benefit. It should have been stated that vision was perfect from the first, and still is so. The lady's circumstances are now less comfortable than formerly, and her habits more sedentary. Yet her health is good, though acupuncture does not now, as formerly, relieve. The attacks are, however, more rare.—*Ibid, from Dublin Med. Press*, No. clxiv., Feb. 23, 1842.

